# Using Fitness Trackers and Smartwatches to Measure Physical Activity in Research: Analysis of Consumer Wrist-Worn Wearables

**DOI:** 10.2196/jmir.9157

**Published:** 2018-03-22

**Authors:** André Henriksen, Martin Haugen Mikalsen, Ashenafi Zebene Woldaregay, Miroslav Muzny, Gunnar Hartvigsen, Laila Arnesdatter Hopstock, Sameline Grimsgaard

**Affiliations:** ^1^ Department of Community Medicine University of Tromsø – The Arctic University of Norway Tromsø Norway; ^2^ Department of Computer Science University of Tromsø – The Arctic University of Norway Tromsø Norway; ^3^ Norwegian Centre for E-health Research University Hospital of North Norway Tromsø Norway; ^4^ Spin-Off Company and Research Results Commercialization Center 1st Faculty of Medicine Charles University in Prague Prague Czech Republic; ^5^ Department of Health and Care Sciences University of Tromsø – The Arctic University of Norway Tromsø Norway

**Keywords:** motor activity, physical activity, fitness trackers, heart rate, photoplethysmography

## Abstract

**Background:**

New fitness trackers and smartwatches are released to the consumer market every year. These devices are equipped with different sensors, algorithms, and accompanying mobile apps. With recent advances in mobile sensor technology, privately collected physical activity data can be used as an addition to existing methods for health data collection in research. Furthermore, data collected from these devices have possible applications in patient diagnostics and treatment. With an increasing number of diverse brands, there is a need for an overview of device sensor support, as well as device applicability in research projects.

**Objective:**

The objective of this study was to examine the availability of wrist-worn fitness wearables and analyze availability of relevant fitness sensors from 2011 to 2017. Furthermore, the study was designed to assess brand usage in research projects, compare common brands in terms of developer access to collected health data, and features to consider when deciding which brand to use in future research.

**Methods:**

We searched for devices and brand names in six wearable device databases. For each brand, we identified additional devices on official brand websites. The search was limited to wrist-worn fitness wearables with accelerometers, for which we mapped brand, release year, and supported sensors relevant for fitness tracking. In addition, we conducted a Medical Literature Analysis and Retrieval System Online (MEDLINE) and ClinicalTrials search to determine brand usage in research projects. Finally, we investigated developer accessibility to the health data collected by identified brands.

**Results:**

We identified 423 unique devices from 132 different brands. Forty-seven percent of brands released only one device. Introduction of new brands peaked in 2014, and the highest number of new devices was introduced in 2015. Sensor support increased every year, and in addition to the accelerometer, a photoplethysmograph, for estimating heart rate, was the most common sensor. Out of the brands currently available, the five most often used in research projects are Fitbit, Garmin, Misfit, Apple, and Polar. Fitbit is used in twice as many validation studies as any other brands and is registered in ClinicalTrials studies 10 times as often as other brands.

**Conclusions:**

The wearable landscape is in constant change. New devices and brands are released every year, promising improved measurements and user experience. At the same time, other brands disappear from the consumer market for various reasons. Advances in device quality offer new opportunities for research. However, only a few well-established brands are frequently used in research projects, and even less are thoroughly validated.

## Introduction

### Background

The World Health Organization recommends 150 min of moderate intensity physical activity (PA) each week for adults and 60 min for children and adolescents [1]. However, 25% of adults and more than 80% of adolescents do not achieve the recommended PA targets [1]. Results from the Tromsø Study, the longest running population study in Norway, shows that only 30.4% of women and 22.0% of men reach the recommended target [2].

Low PA is currently the fourth leading risk factor for mortality worldwide [3]. Even though there is limited evidence that using wearable fitness trackers will improve health [4,5], these devices are still popular, and new fitness devices appear on the consumer market regularly. In 2016, vendors shipped 102 million devices worldwide, compared with 82 million in 2015 [6]. Fifty-seven percent of these devices were sold by the top five brands: Fitbit, Xiaomi, Apple, Garmin, and Samsung. The first quarter of 2017 shows an increase of 18% in devices sold, compared with the same period in 2016 [7]. With a large number of available devices and brands, it is difficult to navigate through an ever-growing list of brands and devices with different capabilities, price, and quality.

Available sensors and internal interpreting algorithms determine device output. Sensor data are, in most devices, reduced to a limited set of metrics before being transferred to the user’s mobile phone. In addition, limited space affects how long the device can collect data before such a transfer is needed. Data are stored locally, and in many cases, uploaded to brand specific or open cloud–based health repositories. Accessing these data by third-party apps and comparing them is not always possible. These interoperability challenges were recently identified in a study by Arriba-Pérez et al [8]. They suggested ways to handle these issues, but they did not make any brand or device recommendations. Several studies have compared activity-tracking wearables. As an example, Kaewkannate and Kim [9] did a comparison of four popular fitness trackers in 2016. They compared devices objectively and subjectively. Data were thoroughly collected, but because of the rapid release of new devices, these four devices will be among the most popular only for a relatively short time. A comparison of brands is also of interest because brands from larger companies are, compared with small start-ups and crowd funded brands, likely to survive longer. In addition, it is of interest to know which brands support the various available programming options. Sanders et al [10] did a literature review on articles using wearables for health self-monitoring and sedentary behavior and PA detection. They reviewed various aspects of these devices, but they gave no details about device sensor support and suitability in research.

The objective of this study was to examine how the consumer market for wearables has evolved, and analyze and summarize available devices that can measure PA and heart rate (HR). Moreover, we aim to identify brands that are used extensively in research projects, and compare and consider their relevance for future studies.

### Sensors

A plethora of devices promises to measure PA in new and improved ways. These devices use different sensors and algorithms to calculate human readable metrics based on sensor output. Traditional step counters use pedometers to detect daily step counts. Although cheap and energy efficient, pedometers are not as accurate as accelerometers, which is the current standard for collecting PA data [11]. All modern fitness trackers and smartwatches have an accelerometer. Compared with research tools (eg, ActiGraph [12]), these devices are considered less accurate for some measurements [13,14]. However, they are generally less invasive, cheaper, have more functionality, are more user-friendly, and are increasingly being used in research. Most accelerometer-based fitness wearables measure acceleration in three directions [15] and can be used to estimate type of movement, count steps, calculate energy expenditure (EE) and energy intensity, as well as estimate sleep patterns and more. The validity and reliability of these metrics varies. Evenson et al [14] did a review in 2015 and found high validity for steps but low validity for EE and sleep. Furthermore, they found reliability for steps, distance, EE, and sleep to be high for some devices.

In addition, some wearables have gyroscopes, magnetometers, barometers, and altimeters. A gyroscope can potentially increase device accuracy by measuring gravitational acceleration, that is, orientation and angular velocity, and better estimate which activity type a person is performing [16]. A magnetometer is a digital compass [15] and can improve motion tracking accuracy by detecting the orientation of the device relative to magnetic north. Magnetometers improve accuracy by compensating for gyroscope drift, a problem with gyroscopes where the rotation axis slowly drifts from the actual motion and must be restored regularly. Accelerometers, gyroscopes, and magnetometers are often combined into an inertial measurement unit (IMU). Most mobile phones use IMUs to calculate orientation, and an increasing number of fitness wearables include this unit to give more accurate metrics. Barometers or altimeters detect changes in altitude [15] and can be used to improve some metrics (eg, EE), as well as report additional metrics (eg, climbed floors).

Photoplethysmography (PPG) is a relatively new technique in wearables. PPG is an optical technique to estimate HR by monitoring changes in blood volume beneath the skin [17]. A light-emitting diode projects light onto the skin, which is affected by the HR and reflected back to the sensor. However, movement, ambient light, and tissue compression affect the light, resulting in signal noise, and cleaning algorithms often use accelerometer data to assist HR estimation [18]. There is some evidence that gyroscopes could be used [19] to reduce PPG signal noise, so we are likely to see more devices in the future equipped with PPG sensors. To further enrich the PA data collection, some devices have a built in global positioning system (GPS) receiver. This is especially true for high-end fitness trackers and sports watches specifically targeting physically active people. With a GPS, it is possible to track more data, including position, speed, and altitude.

### Algorithms and Mobile Apps

Raw data from sensors must be converted into readable metrics to be meaningful for the user. Many devices only display a limited set of metrics directly on the device (eg, today’s step count or current HR) and rely on an accompanying mobile app to show the full range of available metrics (eg, historic daily step count and detailed HR data). Although the physical sensors in these devices are very similar, the algorithms that interpret sensor output are unique for most vendors. These algorithms are often company secrets, and they can be changed without notice. In addition, the quality and supported features of the accompanying mobile apps varies, and the total user experience will therefore differ. Each additional sensor included in a device can be used to add additional types of metrics for the user or supply internal algorithms with additional data to improve accuracy of already available metric types. However, additional sensors affect price and power consumption.

### Device Types

There are many similarities between different types of devices, and they may be difficult to categorize. We will use the term wearable in this paper as a common term for wrist-worn devices that can track and share PA data with a mobile phone.

A smartwatch is a wrist-worn device that, mostly, acts as an extension to a mobile phone and can show notifications and track PA and related metrics. Modern smartwatches often include a touch screen and can support advanced features and display high resolution activity trends [15]. Fitness trackers (ie, smart band or fitness band), normally worn on the wrist or hip, are devices more dedicated to PA tracking. A fitness tracker is typically cheaper than a smartwatch because of less expensive hardware and often fewer sensors. Due to this, it generally also has better battery life and a limited interface for displaying tracking results [15].

Other terms are also used, for example, sports watch and GPS watch, which can be considered merges between smartwatches and fitness trackers. In addition, there are hybrid watches (ie, hybrid smartwatches) that have a traditional clockwork and analogue display that have been fitted with an accelerometer. An accompanying mobile app is needed to access most data, but daily step counts are often represented as an analogue gauge on the watch face.

### Wearable Usage Scenario

Wearables come forward as a new alternative to tracking PA in research (compared with, eg, ActiGraph), especially when it is desired to collect measurements for a prolonged period of time. In an intervention study, continuous data collecting from wearables would allow researchers to better track changes in PA and adjust the intervention accordingly. Wearables can also be used in epidemiological research as a tool for tracking PA for an extended period. This could reveal detailed PA changes in a population over time. In both scenarios, there are several potential important requirements to consider when choosing a device for the study, including usability, battery life, price, accuracy, durability, look and feel, and data access possibilities.

## Methods

### Search Strategies

#### Brands, Devices, and Sensors

We searched six databases to create a list of relevant wearable devices: The Queen’s University’s Wearable Device Inventory [20], The Vandrico Wearables database [21], GsmArena [22], Wearables.com [23], SpecBucket [24], and PrisGuide [25,26]. We only used publicly available information when comparing devices. We did the search from May 15, 2017 to July 1, 2017.

We identified wearables in two steps. In step one, we identified and searched the six defined databases. In step two, we extracted all brands from the list of devices identified in step one and examined brand websites for additional devices. If we found the same device in several databases with conflicting information, we manually identified the correct information from the device’s official website or other online sources (eg, Wikipedia and Google search). We removed duplicates and devices not fitting the inclusion criteria.

#### Brand Usage in Research

We searched Ovid MEDLINE on September 30, 2017 to determine how often the most relevant brands were used in previous studies. For each search, we performed a keyword search with no limitations set. We divided our findings into validation and reliability studies and data collection studies.

To decide which brand to consider most relevant, we did two sets of searches. In the first set, we created a brand-specific keyword search for brands that were (1) One of the five most sold brands in 2015 or 2016 or (2) Had released 10 or more unique devices. From the resulting list of articles, we screened title, abstract, and the method section. This screening was done to (1) Exclude articles out of scope and (2) To identify additional brands used in these studies. We compiled a list of these brands and performed a second set of searches, one for each new identified brand. Eleven brands were finally included. The specific keyword search used for each brand is given in the Results section where we summarize our findings.

We also searched the US National Library of Medicine database of clinical studies through the ClinicalTrials website, using the same 11 keyword searches, to determine brand usage in ongoing projects. One author did the articles screening, as well as the projects description screening in ClinicalTrials.

#### Brand Developer Possibilities

To determine how relevant a specific brand is when planning a new research project, we reviewed the 11 identified brands and considered available developer options, supported mobile phone environments, and options for health data storage. We especially reviewed availability of an application programming interface (API) and a software development kit (SDK). Information was collected from Google Play, Apple’s App Store, and official brand websites. Information retrieval was done in September 2017.

### Inclusion and Exclusion Criteria

#### Brands, Devices, and Sensors

The study is limited to wrist-worn consumer devices that utilize accelerometers to measure PA. Devices capable of collecting HR from the wrist using an optical sensor were tagged as PPG devices. Devices were tagged as GPS devices only if they had a built-in GPS tracker. We only included devices meant for personal use, designed to be worn continuously (24/7), and were capable of sharing data with mobile phones through Bluetooth. The wrist-worn limitation was added because hip-worn devices are not normally worn during the night (ie, not 24/7). Only devices released before July 1, 2017 were included. We excluded hybrid watches because most hybrid vendors make a large number of watch variations, with what seems to be the same hardware. In addition, these watches are mostly available through high-end suppliers of traditional watches, at a price point that would prevent researchers from considering their use in a large study.

#### Brand Usage in Research

Due to the large number of available brands, we limited our search to include only the 11 brands already identified as relevant. We excluded brands that are no longer available (ie, company shut down). Review studies were also excluded.

#### Brand Developer Possibilities

When reviewing brand relevance in research, we only reviewed developer capabilities for the 11 brands we had already included in the list of relevant brands. We set the additional limitation that the brand was used in at least one article in Ovid MEDLINE.

### Device Categorization, Data Collection, and Reporting Categories

When collecting information about wearables, we categorized them into three groups:

1. Smartwatches: a device was tagged as a smartwatch if
  - It supported mobile phone notifications, and the vendor described it as a smart watch, or if
  - It had a touch screen and was not explicitly described as a fitness tracker by the vendor.
2. Fitness trackers: we classified a device as a fitness tracker if
  - Its main purpose was to track PA, or if
  - The vendor called it a fitness tracker, or if
  - The device did not support notifications from the connected mobile phone (eg, incoming calls or texts).
3. Hybrid watches: to be considered a hybrid watch, the device had to have an analogue clockwork with a built-in digital accelerometer.

We collected the following variables for each device: brand name, device name, year of release, country of origin, device type (eg, fitness tracker), and whether they had a built-in accelerometer, gyroscope, magnetometer, barometer or altimeter, GPS, and PPG.

We looked at three aspects of the devices we identified and reported under three categories:

1. Metrics and trends: in this category, we described the status for available brands, devices, and sensors, as well as reviewed trends in sensor availability over time.
2. Brand usage in research: in this category, we searched Ovid MEDLINE and ClinicalTrials and determined which brands are most used in a research setting.
3. Brand developer possibilities: in this category, we reviewed software integration platforms and mobile platform support for the most relevant brands.

## Results

### Relevant Devices

**Figure 1:**
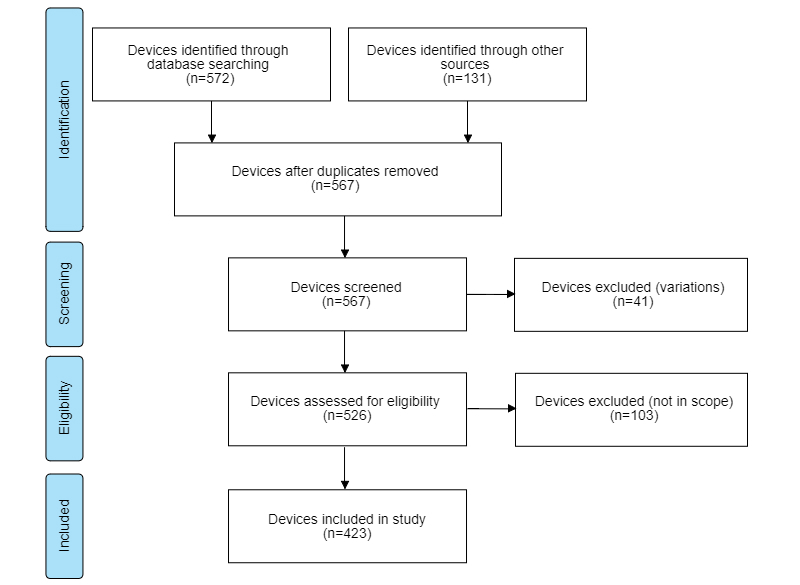
Preferred Reporting Items for Systematic Reviews and Meta-Analyses (PRISMA) flowchart.

An overview of the device search process is given in Figure 1. We found 572 devices by searching online and offline databases and 131 additional devices by visiting the official websites for each identified brand, totaling 703 devices. Removing duplicates left 567 unique devices. These were screened for variation, that is, the same device with different design. After excluding 41 because of variation, 526 remained and were screened for eligibility. We removed 103 devices for not fitting the inclusion criteria. The remaining 423 devices were included in the study.

### Brands, Devices, and Sensors

#### Brands

We identified 423 unique wearables, distributed between 132 different brands. Almost half the brands (47.0%, 62/132) had only one device. Moreover, 75.0% (99/132) of brands had three or fewer devices, and 83.3% (110/132) had five or fewer devices. Brands originated from 23 different countries, but the United States (43.2%, 57/132) and China (16.7%, 22/132, mainland China; 19.0%, 25/132, including Taiwan) represented the largest number of brand origin. Each remaining country represented between 0.8% (1/132) and 5.3% (7/132) of brands.

**Figure 2:**
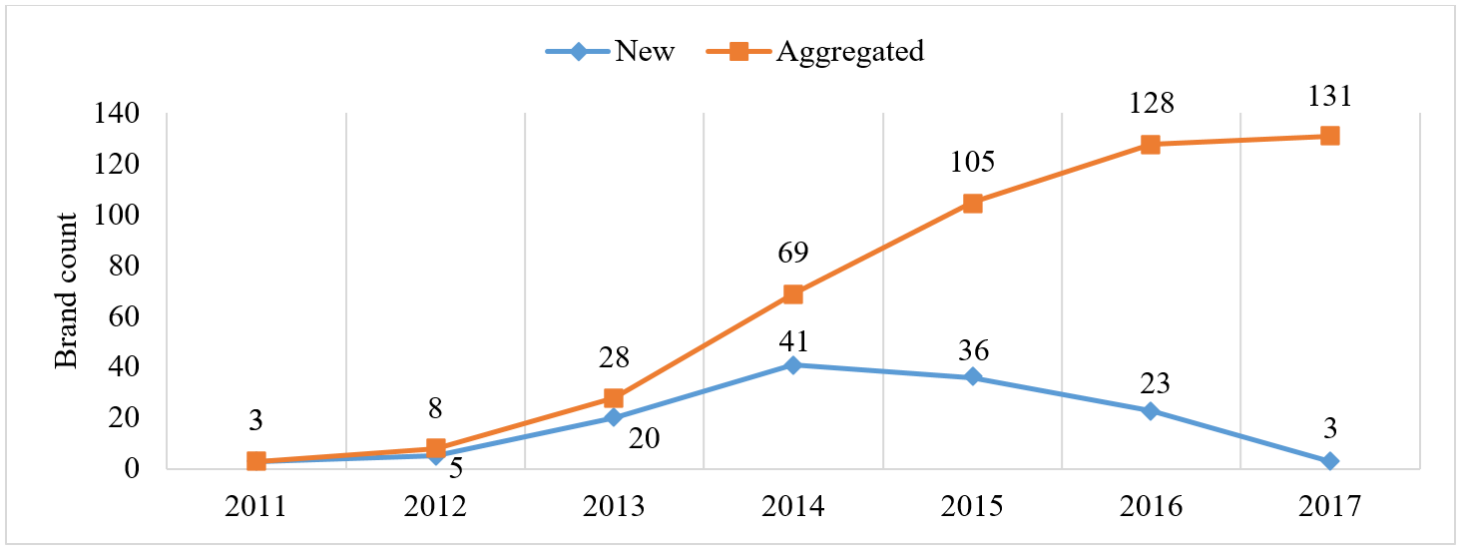
Number of new and aggregated available brands by year.

As the market has grown and wearable technology has become increasingly popular, a number of new brands have appeared on the market. In 2011, there were only three brands available. There was a small increase in brand count in 2012 and 2013, but in 2014, we saw the largest increase with 41 new brands. The number of new brands started to decrease in 2015, with 36 new brands in 2015 and 23 in 2016. Only three new brands have been introduced in 2017, but this number only represents the first 6 months of 2017. The final count for 2017 will likely be higher. An overview of the number of new brands that appeared on the market between 2011 and 2017 is given in Figure 2. Note that some companies are no longer active and, for 17 devices, we could not determine release year.

Most brands only had a small number of wearables, but some produced a lot more. The brand with most unique wearables was Garmin (United States) with 40 different devices. No.1 (China) introduced the second highest number of wearables with 19 devices. An overview of the release year of the 22 (out of 132) brands that have released more than five devices is given in Table 1. Seven out of these 22 brands originated in the United States, five (six including Taiwan) originated in China, and two originated in South Korea. All other countries are represented only once. Some of these brands are no longer active (eg, Pebble and Jawbone).

#### Devices

Three devices were released in 2011 (earliest year), seven in 2012, 30 in 2013, and 87 in 2014. The year with the highest number of new wearables was 2015, with 121 new devices. In 2016, 120 new devices were released; the first year with a decreasing number of new wearables. The number of new and accumulated devices from 2011 to 2017 is summarized in Table 2. The last column (unknown) represents devices where we could not identify the release year. The above numbers represent the total number of new devices. If grouped into fitness trackers and smartwatches, there is a small overrepresentation among new smartwatches. Up until 2014, about half of devices were smartwatches. In 2015 and 2016, smartwatches represented 59.3% (143/241) of new devices, whereas fitness trackers represented 40.6% (98/241).

#### Sensors

**Figure 3:**
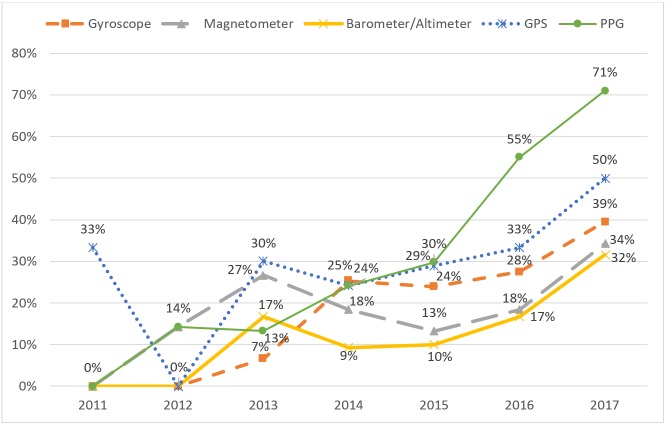
Percentage of devices released each year, supporting each sensor. GPS: global positioning system; PPG: photoplethysmography.

The number of sensors included in new devices have increased in the last few years. Since 2015, the order of the most common sensors has consistently been PPG, GPS, gyroscope, magnetometer, and barometer or altimeter. In addition, these sensors have had a steady increase in availability in the same period. For 2017, 71% (27/38) of new devices included a PPG sensor, 50% (19/38) included a GPS, 39% (15/38) included a gyroscope, 34% (13/38) included a magnetometer, and 32% (12/38) included a barometer or altimeter. Figure 3 gives an overview of the number of devices each year that includes each sensor, in percent of total number of released devices that year. Devices with more than one sensor are represented once for each sensor it includes.

In total, since 2011, 38.5% (163/423) of wearables have only been equipped with one sensor (accelerometer). Moreover, 29.8% (126/423) of devices had two sensors, 12.1% (51/423) had three sensors, 11.1% (47/423) had four sensors, and 6.4% (27/423) had five sensors. Only 2.1% (9/423) of devices had all six sensors. In Table 3, these numbers are broken down by sensor combination and year. Some sensor combinations do not exist and are excluded.

**Table 1:** Device count per year for brands with six or more wearables.

Brand	Country	2011	2012	2013	2014	2015	2016	2017	Unknown	Total^a^
Garmin	United States		1	5	6	11	13	4		40
Fitbit	United States			1	1	2	4	1		9
Misfit	United States			1	1	3	1	2		8
LifeTrak	United States			1	5		1			7
iFit	United States				1	4	1			6
Jawbone	United States	1		1	1	3				6
Pebble	United States			1	1	3	1			6
No. 1	China					5	9	5		19
Omate	China				2	5	2			9
Zeblaze	China					2	5	2		9
Huawei	China				1	3	3	1		8
Oumax	China				1	2	2	1	1	7
Mobile Action	Taiwan					2	2		4	8
Samsung	South Korea			1	6	1	4			12
LG	South Korea				3	1	1	2		7
WorldSim	England					1	1		5	7
Polar	Finland			1	2	4	2	2		11
Technaxx	Germany				4		2			6
Awatch	Italy						3	4		7
Epson	Japan				2	5				7
TomTom	Netherlands				2	1	4			7
MyKronoz	Switzerland				4	6	7	1		18

^a^Total brand count for the United States=7, China and Taiwan=6, and South Korea=2. All other countries are represented only once.

**Table 2:** Number of new and accumulated devices by year.

Devices	2011	2012	2013	2014	2015	2016	2017	Unknown
New	3	7	30	87	121	120	38	17
Accumulated	3	10	40	127	248	368	406	423

### Brand Usage in Research

The top five vendors in 2015 [27] and 2016 [6], in sold units, were Fitbit, Xiaomi, Apple, Garmin, and Samsung. Brands with more than 10 unique wearables include Garmin, No.1, MyKronoz, Samsung, and Polar. These eight, and additional brands identified during the MEDLINE search and ClinicalTrials search, were considered. We did not find any publications or active clinical trials that used devices from No.1 or MyKronoz. Devices from Basis, BodyMedia, Pebble, Jawbone, Microsoft, and Nike were also used in some of the identified studies, but these brands do no longer produce wearables within the scope of this paper and were excluded from further analysis.

The MEDLINE search resulted in 81 included studies that we divided into two groups: (1) validation and reliability studies and (2) data collection studies. Studies where wearable output was compared with existing research instruments known to give accurate results (eg, ActiGraph) or with direct observation, as well as studies where several wearables were compared with each other for accuracy or reliability, were classified as validation and reliability studies. Studies where wearables were used as a tool for intervention or observation, to collect data on PA, HR, EE, sleep, or other available metrics, were classified as data collection studies. Out of these 81 studies, 61 were classified as validation and reliability studies, whereas 20 were classifies as data collection studies.

Fitbit devices were used in 54 studies [9,13,28-79]. Out of these, 40 studies were validation or reliability studies. In 22 of the studies, one or more Garmin devices were used [32,33,46,49,50,62,77-92]. Of these, 18 were validation or reliability studies. Eight studies used Apple devices [29,30,35,49,62,79,93,94]. Six of these were validation or reliability studies. All studies using devices from Misfit, Polar, Withings, Mio, Samsung, PulseOn, TomTom, and Xiaomi were validation or reliability studies. Misfit devices were used in 12 studies [9,36,42,43,46,61-63,85,95-97]; Polar devices were used in 6 studies [36,43,46,62,98,99]; Withings [63,85,89,100,101], Mio [29,30,54,102,103], and Samsung [29,30,58,62,96] devices were used in 5 studies; PulseOn devices were used in 4 studies [29,104-106]; TomTom devices were used in 2 studies [54,79]; and Xiaomi devices were used in 1 study [96].

From ClinicalTrials, we found that the vast majority of ongoing projects use, or are planning to use, Fitbit devices. All other devices were mentioned in three or less projects, whereas Fitbit devices were mentioned in 31 studies. A summary of these studies and projects is given in Table 4. We further grouped the validation and reliability studies into five categories. A total of 31 studies focused on step counts or distance, 15 studies researched EE, 15 studies measured HR, 10 studies measured sleep, and 7 studies collected other metrics. Multimedia Appendix 1 gives an overview of articles found in MEDLINE, which brands they included in the study, and which of the five categories they are grouped into.

### Brand Developer Possibilities

Next, we considered developer possibilities for the 11 brands already identified as most relevant in research: Apple, Fitbit, Garmin, Mio, Misfit, Polar, PulseOn, Samsung, TomTom, Withings, and Xiaomi. All brands had an app in the Apple App Store and could connect to the iPhone. Except for the Apple Watch, all other brands had an app in Google Play and could be used with Android phones.

**Table 3:** Number and percentage of devices supporting a specific group of sensors, by year.

Sensors		2011	2012	2013	2014	2015	2016	2017
Accelerometer (Acc), n (%)		2 (67)	5 (71)	16 (53)	40 (46)	50 (41.3)	37 (30.8)	4 (11)
**Acc + 1 sensor, n (%)**								
	PPG^a^		1 (14)	1 (3)	9 (10)	11 (9.1)	27 (22.5)	10 (26)
	GPS^b^	1 (33)		2 (7)	9 (10)	15 (12.4)	3 (2.5)	
	Gyroscope (Gyro)			1 (3)	3 (3)	9 (7.4)	4 (3.3)	1 (3)
	Magnetometer (Mag)		1 (14)	2 (7)	1 (1)	3 (2.5)		
	Barometer (Bar)				1 (1)	1 (0.8)		2 (5)
**Acc + 2 sensors, n (%)**								
	GPS + PPG			1 (3)		7 (5.8)	6 (5)	3 (8)
	Gyro + PPG				4 (5)	5 (4.1)	5 (4.2)	1 (3)
	Gyro + GPS				1 (1)	2 (1.7)	2 (1.7)	
	Bar + PPG			1 (3)		1 (0.8)	2 (1.7)	
	Gyro + Mag				2 (2)	1 (0.8)		
	Mag + GPS			1 (3)	1 (1)	1 (0.8)		
	Mag + PPG						1 (0.8)	
	Gyro + Bar				1 (1)			
	Bar + GPS				2 (2)			
**Acc + 3 sensors, n (%)**								
	Gyro + Mag + GPS			1 (3)	3 (3)	3 (2.5)	2 (1.7)	1 (3)
	Gyro + Mag + PPG				4 (5)	2 (1.7)	3 (2.5)	1 (3)
	Mag + Bar + GPS			3 (10)	2 (2)		4 (3.3)	1 (3)
	Gyro + GPS + PPG				1 (1)		6 (5)	1 (3)
	Bar + GPS + PPG						2 (1.7)	2 (5)
	Mag + GPS + PPG						1 (0.8)	1 (3)
	Gyro + Bar + PPG					2 (1.7)		
	Gyro + Mag + Bar						1 (0.8)	
**Acc + 4 sensors, n (%)**								
	Mag + Bar + GPS + PPG			1 (3)		3 (2.5)	4 (3.3)	
	Gyro + Mag + GPS + PPG				1 (1)		3 (2.5)	3 (8)
	Gyro + Bar + GPS + PPG					2 (1.7)	4 (3.3)	1 (3)
	Gyro + Mag + Bar + GPS						1 (0.8)	2 (5)
	Gyro + Mag + Bar + PPG				1 (1)	1 (0.8)		
**Acc + 5 sensors, n (%)**								
	All sensors				1 (1)	2 (1.7)	2 (1.7)	4 (11)
Total, n		3	7	30	87	121	120	38

^a^PPG: photoplethysmography.

^b^GPS: global positioning system.

**Table 4:** Number of identified articles in Medical Literature Analysis and Retrieval System Online (MEDLINE) and ClinicalTrials.

Brand	MEDLINE^a^ search term	MEDLINE		ClinicalTrials	
		Validation or reliability studies^b^ (total article count=61)	Data collection studies^c^ (total article count=20)	Validation or reliability studies^d^	Data collection studies^e^
Fitbit	Fitbit AND (Alta OR Blaze OR Charge OR Flex OR Surge)	40	14	1	30
Garmin	Garmin AND (Approach OR D2 OR Epix OR Fenix OR Forerunner OR Quatix OR Swim OR Tactix OR Vivo*)	18	4	1	2
Misfit	Misfit AND (Flare OR Flash OR Link OR Ray OR Shine OR Vapor)	12	0	0	1
Apple	Apple watch	6	2	1	1
Polar	Polar AND (“Polar Loop” OR M200 OR M4?0 OR M600 OR V800 OR A3?0)	6	0	1	3
Withings	Withings	5	0	0	2
Mio	Mio Alpha OR Mio Fuse OR Mio Slice	5	0	1	2
Samsung	Samsung Gear NOT “Gear VR” NOT Oculus	5	0	0	2
PulseOn	PulseOn	4	0	0	1
TomTom	TomTom	2	0	1	
Xiaomi	Xiaomi	1	0	0	1

^a^MEDLINE: Medical Literature Analysis and Retrieval System Online.

^b^Number of validation or reliability studies in MEDLINE.

^c^Number of data collection studies in MEDLINE.

^d^Number of validation or reliability studies in ClinicalTrials.

^e^Number of data collection studies in ClinicalTrials.

Three brands supported Windows Phone: Fitbit, Garmin, and Misfit. Apple Health and Google Fit are the two most common open cloud health repositories. Mio, Misfit, Polar, Withings, and Xiaomi, were the only brands that automatically synchronized fitness data to both of these repositories through these open APIs. The Apple Watch only synchronized automatically to the Apple Health repository. Seven out of 11 brands had a private cloud repository with an accompanying API, which allows third-party apps to access these data. Five brands had an SDK, which makes it possible to create custom programs to communicate with the device or create watch faces that can run on the device.

The Apple Watch was the only device running on watchOS. Three brands had at least one device running on Android Wear. The remaining seven brands used a custom system. A summary of all attributes for each brand is given in Table 5. Not all devices for a specific brand support all features. In addition, this is a snapshot of the status of these attributes, which are likely to change over time as new devices and brands expand their capabilities. The Apple Watch development environment is called WatchKit SDK and can be used to write apps for the Apple Watch [107]. Apple’s health storage solution is called Apple Health. A variety of different data types can be stored here and accessed by third-party developers through the HealthKit API [108]. Access to any of these services requires enrollment in the Apple Developer Program, which currently costs US $99 per year.

Fitbit offers three major SDKs (Device API, Companion API, and Settings API) for developing apps for Fitbit devices. In addition, Fitbit offers the Web API that can be used to access Fitbit cloud-stored fitness data. The Web API exposes six types of data: PA, HR, location, nutrition, sleep, and weight [109]. Fitbit also has a solution for accessing high-resolution step and HR data (ie, intraday data), granted on a case by case basis. There is no cost for developing with the Fitbit SDKs or API.

There are two generations of programmable Garmin wearables [110]. The Connect IQ SDK can be used by both generations, but devices using the newer Connect IQ 2 generation support more features. Development with this SDK is free. Garmin also offers a cloud-based Web API, Garmin Connect, which allows third-party apps to access users’ cloud-based fitness data. Access to this API costs US $5000 (one-time license). In addition, Garmin maintains a separate Health API intended to be used by companies for wellness improvement of their employees. This API is free but requires a manual approval from Garmin.

**Table 5:** Brand environment, integration, and development support.

Feature		Apple	Fitbit	Garmin	Mio	Misfit	Polar	PulseOn	Samsung	TomTom	Withings	Xiaomi
**Supported platform**												
	Android		✓	✓	✓	✓	✓	✓	✓	✓	✓	✓
	iPhone	✓	✓	✓	✓	✓	✓	✓	✓	✓	✓	✓
	Windows phone		✓	✓		✓						
**Integration**												
	Automatic synchronization to Apple Health	✓			✓	✓	✓				✓	✓
	Automatic synchronization to Google Fit				✓	✓	✓				✓	✓
	Private cloud storage		✓	✓		✓	✓		✓	✓	✓	
	Cloud storage API^a^	✓	✓	✓		✓	✓		✓	✓	✓	
	Developer SDK^b^	✓	✓	✓		✓			✓			
**Watch system**												
	Android Wear					✓	✓					✓
	watchOS (Apple)	✓										
	Custom		✓	✓	✓			✓	✓	✓	✓	

^a^API: application programming interface.

^b^SDK: software development kit.

The Misfit developer ecosystem consists of three SDKs (Sleep SDK, Link SDK, and Device SDK) [111]. The Misfit Device SDK is the major SDK for developing apps for and communication with Misfit devices. This SDK is only available on request. Misfit also offers the Misfit Scientific Library that can be used to access Misfits proprietary sensor algorithms directly. This library is also only available on request. In addition, the Misfit Cloud API is used to access users’ data from the Misfit cloud server. All SDKs and the API are free.

Polar does not offer a separate SDK. Polar devices can integrate with Google Fit and Apple Health and deposits collected data there [112]. This data are accessed using Google Fit APIs and Apple HealthKit APIs. In addition, data are uploaded to Polar’s cloud storage, which is accessible by third-party developers through the AccessLink API. Besides PA data (steps, EE, and sleep), basic training data are also stored here. Access to AccessLink is free.

Development for a Samsung smartwatch is done using the Tizen SDK (Samsung smartwatch operating system is called Tizen). The Samsung Health SDK platform consists of two parts: Data SDK and Service SDK. Together these can be used to store and access health data collected from internal and external sensors, as well as third-party apps running on a Samsung watch or a mobile phone. Development using any of these services is free [113].

TomTom offers the Sports Cloud API for accessing data collected from TomTom devices. The API provides four types of data: PA (eg, exercises bouts), HR, tracking (eg, steps and EE), and physiology (eg, weight). Access to the API is free [114].

Nokia acquired Withings in 2016, and the original Withings API is now available as the Nokia Health API. Besides PA and sleep measurements, the API also gives access to intraday PA data. Nokia must manually approve access to this high-resolution activity API. The API is free [115].

### Summarizing Results

Which features are most important when considering devices for a research project will depend on the purpose and design of the study. It is therefore not possible to identify one brand as the best brand in all circumstances. However, we have tried to quantify various aspects of a brand to identify and summarize their benefits.

**Table 6:** Brand summary.

Brand		Fitbit	Garmin	Misfit	Apple	Polar	Samsung	Withings	Mio	PulseOn	TomTom	Xiaomi	MyKronoz	No. 1
Devices^a^		9	40	8	3	11	12	2	3	1	7	3	18	19
MEDLINE^b^		54	22	12	8	6	5	5	5	4	2	1		
**Validation or reliability^c^**		40	18	12	6	6	5	5	5	4	2	1		
	Steps	21	10	6	1	2	2	4				1		
	Energy expenditure	10	4	3	4	3	1		2	2				
	Heart rate	7	4	1	4	1	2		5	4	2	1		
	Sleep	8	1	4		1		2						
	Other	3	4	2		1								
ClinicalTrials^d^		31	3	1	2	4	2	2	3	1	1	1		
SDK^e^		✓	✓	✓	✓	✓	✓							
API^f^		✓	✓	✓	✓	✓	✓	✓			✓			
Apple Health^g^				✓	✓	✓		✓	✓			✓		
Google Fit^h^				✓		✓		✓	✓			✓		

^a^Number of unique devices.

^b^MEDLINE: Medical Literature Analysis and Retrieval System Online. Number of articles in MEDLINE.

^c^Number of validation or reliability studies in MEDLINE, grouped by metric (step, EE, HR, sleep, and others).

^d^Number of active projects in ClinicalTrials.

^e^Supports an SDK for third-party software implementation.

^f^API: application programming interface. Supports an API for developer access to data cloud.

^g^Supports automatic synchronization to Apple Health data cloud.

^h^Supports automatic synchronization to Google Fit data cloud.

We used eight categories in this custom comparison, which we suggest to consider before deciding on a brand for any research project:

1. Device count: a higher number of available devices make it possible to pick a device that is more tailored to the study.
2. Article count: a higher number of articles in Ovid MEDLINE indicate usage in previous studies.
3. Validation or reliability count: a high number of validation or reliability studies provides knowledge about device and brand accuracy.
4. ClinicalTrials count: a high number of active projects in ClinicalTrials indicate brand relevance.
5. SDK support: brands that allows third-party programs to run on their devices or communicate directly with the device, by offering an SDK, adds more possibilities for customization.
6. API support: brands that allows third-party programs to access the data cloud repository, by offering API access, adds more possibilities for health data collection and retrieval.
7. Apple Health: brands supporting automatic synchronization to Apple Health allow usage of Apple HealthKit API.
8. Google Fit: brands supporting automatic synchronization to Google Fit allow usage of Google Fit API.

A consensus between authors was reached to include these specific categories because we think together they indicate how often a specific brand has been used in the past and will be used in the future, and they show which options are available for data extraction. These are not the only possible categories, and each category will not be equally important for all studies.

Table 6 gives a summary of these categories for each brand. A transposed Excel (Microsoft) version for dynamic sorting is given in Multimedia Appendix 2. We have divided MEDLINE validation and reliability studies into subgroups, making it easier to compare brands for specific study purposes.

## Discussion

### Availability and Trends

The number of new brands increased every year from 2011 to 2014, but from 2015 to 2016, we saw a decrease in the number of new brands. The number of new devices also increased from 2011 to 2015, with a slight reduction in 2016. Many new and existing companies have tried to enter the wearable market during these years. Some have become popular, whereas others are no longer available. The number of new devices in the first two quarters of 2017 seems low, and there is a small indication that the number of new brands and devices released each year is declining. During the data collection phase, we also identified a large number of hybrid watches. Although we did not report on these, this relatively new branch of wearables has grown in popularity. The Fossil group, representing 19 brands, recently announced they would launch more than 300 hybrid watches and smartwatches in 2017 [116]. Most of these will be hybrids, and 2017 may see the highest number of new hybrids released to date.

We only found nine devices that support all five sensors considered in this study. Among the 11 most relevant brands, only Fitbit Surge, Garmin Forerunner 935, Garmin Quatix 5, Samsung Gear S, and TomTom Adventure fall in this category. Most devices (68%) support only one sensor, in addition to the accelerometer. These numbers indicate that sensor count is not the main argument when choosing a device for personal use. In addition to the accelerometer, the most common sensors are PPG and GPS, regardless of sensor count. One reason for this may be that the added benefit of having these sensors, in a fitness setting, is very clear. Accelerometers can be used for step counting, PA intensity, exercise detection, and other well-understood metrics, whereas the added benefit of a gyroscope may be less intuitive. The added convenience of using a PPG compared with a pulse chest strap, or no HR detection at all, is also easy to understand. Adding a GPS also adds some easy-to-understand benefits, where tracking progress on a map and the possibility to detect speed is the most obvious. Magnetometers and barometers or altimeters may not be sensors that most people consider relevant for PA, although they can be used to enhance accuracy of EE and other metrics.

### Brand Usage in Research

In the MEDLINE literature search, we found 81 studies that used one or more of the 11 brands we identified as most relevant in research. Out of these, 61 were validation or reliability studies. The remaining 20 studies used wearable devices as data collection instruments to measure PA, HR, EE, sleep, or other metrics. Fitbit was used in twice as many validation or reliability studies as any other brand. This has likely contributed to the high number of studies where Fitbit was used as the only instrument for health data collection. The same trend will likely continue in future publications because numbers from ClinicalTrials for active projects shows an overrepresentation of Fitbit-enabled projects. Of the brands currently available, the five most often used in research projects are Fitbit, Garmin, Misfit, Apple, and Polar. In addition, these brands have all existed for several years and have either released a large number of unique devices or shipped a large number of total devices. As such, they are likely to stay on the market for the near future.

A high article count, high number of validation or reliability studies, or high number of studies in ClinicalTrials for a specific brand does not automatically imply validity or reliability. It does, however, show researcher interest in these brands.

### Implication for Practice

Table 6 is a good starting point when considering brands for a new research project. Article count, validation or reliability study count, and ClinicalTrials count together indicate brand dependability. Larger numbers indicate how relevant, usable, and valid previous researchers have found each brand to be. In projects where it is relevant, SDK support allows programmatic interaction directly with the device. API support allows storage in, and access to, a brand-specific cloud-based health data repository. Apple Health and Google Fit support are alternative solutions for storing and accessing health data in an open cloud repository. For projects that require multiple brand support, using open solutions reduces the need to implement specific software for each brand. SDK, API, Apple Health, and Google Fit must be supported on both the brand and device level, however.

A high brand device count makes it easier to find a device that best supports the study needs. In addition to available sensors (ie, metrics), validation, and previous usage in research, several other potential relevant criteria exist, including price, availability, phone environment support, affiliated app features, look and feel, battery life, build quality or robustness, water resistance, connectivity, and usability.

**Figure 4:**
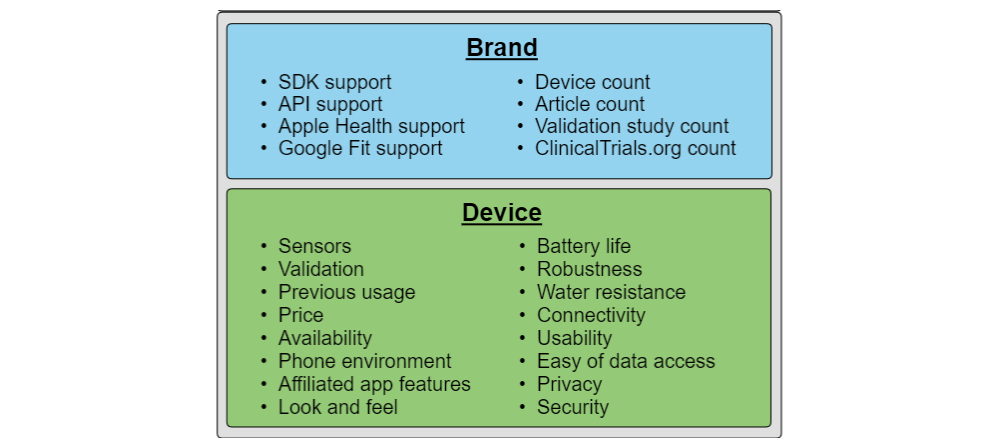
Criteria to consider when choosing brand or device. API: application programming interface; SDK: software development kit.

Furthermore, projects that need programmatic access to the wearable or stored health data should especially consider SDK or API features and ease of use, as well as privacy and security. Figure 4 gives a summary of criteria to consider when selecting brand and device.

### Limitations

We visited all the brands’ websites to find additional devices, but several sites did not contain any information about discontinued devices. The release year of a device was rarely available on device webpages, and we had to search for reviews and other sources to find this information. The level of detail in device hardware specifications varied. Some vendors did not specify which sensor they included in their devices and only mentioned which features the device had. In some cases, the sensor could be derived from this information, but in other cases, we had to find this information elsewhere. Wikipedia was also used to collect sensor support and release year for some devices. This open editable encyclopedia is not necessarily always updated with correct information. For these reasons, there may be some inaccuracies in reported sensor support and release year. We did not collect information about device discontinuation. Reported numbers for total available devices does, therefore, not reflect the numbers of devices that currently can be store bought but rather the number of unique devices that have existed at some point.

### Conclusions

In the last few years, we have seen a large increase in available brands and wearable devices, and more devices are released with additional sensors. However, for activity tracking, some sensors are more relevant than others are. In this study, we have focused on sensor support, health data cloud integration, and developer possibilities; because we find these to be most relevant for collection of PA data in research. However, deciding which wearable to use will depend on several additional factors.

The wearable landscape is constantly changing as new devices are released and as new vendors enter or leave the market, or are acquired by larger vendors. What currently are considered relevant devices and brands will therefore change over time, and each research project should carefully consider which brand and device to use. As a tool for future research, we have defined a checklist of elements to consider when making this decision.

## Appendix

Multimedia Appendix 1 List of MEDLINE articles included in the results for "Brand usage in research".

Multimedia Appendix 2 Summary of the most important categories to consider when selecting a wearable brand for research.

## Abbreviations

API: application programming interface
EE: energy expenditure
GPS: global positioning system
HR: heart rate
IMU: inertial measurement unit
MEDLINE: Medical Literature Analysis and Retrieval System Online
PA: physical activity
PPG: photoplethysmography
SDK: software development kit
